# Supplementary material for: Astrocytic ALKBH5 in stress response contributes to depressive-like behaviors in mice
Source: Nat Commun. 2024 May 21;15:4347. doi: 10.1038/s41467-024-48730-2 (PMC11109195; doi:10.1038/s41467-024-48730-2)
Supplement: Supplementary file 1 — Supplementary Information [file 41467_2024_48730_MOESM1_ESM.pdf]

Supplementary Information

Astrocytic ALKBH5 in stress response contributes to depressive-like behaviors in mice.

Authors: Fang Guo[1#], Jun Fan[2#], Jin-Ming Liu[1#],Peng-Li Kong[1], Jing Ren[1], Jia-Wen Mo[1], Cheng-Lin Lu[1], Qiu-Ling Zhong[1], Liang-Yu Chen[1], Hao-Tian Jiang[1], Canyuan Zhang[1], You-Lu Wen[3], Ting-Ting Gu[3], Shu-Ji Li[1], Ying-Ying Fang[1], Bing-Xing Pan[4], Tian-Ming Gao[1], Xiong Cao[1, 5, 6*]

Affiliations:

[1]Key Laboratory of Mental Health of the Ministry of Education, Guangdong-Hong Kong-Macao Greater Bay Area Center for Brain Science and Brain-Inspired Intelligence, Guangdong-Hong Kong Joint Laboratory for Psychiatric Disorders, Guangdong Province Key Laboratory of Psychiatric Disorders, Guangdong Basic Research Center of Excellence for Integrated Traditional and Western Medicine for Qingzhi Diseases， Department of Neurobiology, School of Basic Medical Sciences, Southern Medical University, Guangzhou, China.

[2]Department of Anesthesia, Guangzhou Women and Children's Medical Center, Guangzhou Medical University, Guangdong Provincial Clinical Research Center for Child Health, Guangzhou, Guangdong, China

[3]Department of Psychology and Behavior, Guangdong 999 Brain Hospital, Institute for Brain Research and Rehabilitation, South China Normal University, Guangzhou, Guangdong, P. R. China

[4]Department of Biological Science, School of Life Science, Nanchang University, Nanchang, China.

[5]Department of Oncology, Nanfang Hospital, Southern Medical University Guangzhou, Guangdong, P. R. China

[6]Microbiome Medicine Center, Department of Laboratory Medicine, Zhujiang Hospital, Southern Medical University, Guangzhou, Guangdong, P. R. China

*e-mail: caoxiong@smu.edu.cn.

Supplementary Figure 1

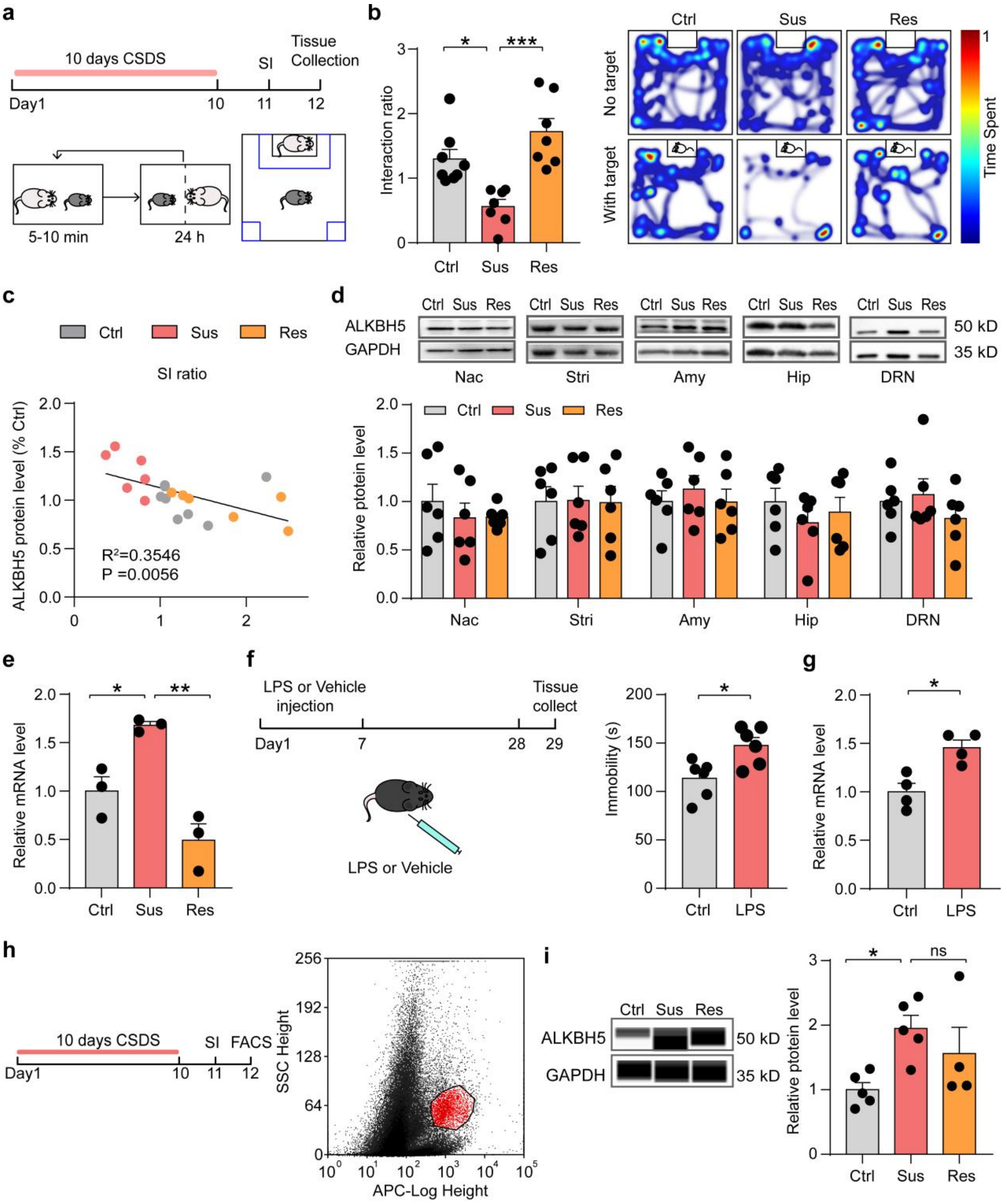

Supplementary Figure 1 the ALKBH5 expression in the mouse models of depression. a-b, Experimental paradigm (**a**) of chronic social defeat stress (CSDS), and Social Interaction (SI) ratio of Sus, Res, and Ctrl mice (**b**). (Ctrl, n=8; Sus, n=6; Res, n=6 mice). **c**, ALKBH5 protein in the mPFC of mice after CSDS correlated with social avoidance. (Ctrl, n=8; Sus, n=6; Res, n=6 mice). **d**, ALKBH5 protein in the Nac, Stri, Amy, Hip, DRN of mice after CSDS. (n=6 mice per group). **e**, qRT-PCR analysis of ALKBH5 mRNA in the mPFC of female mice after CSDS. (n=3 mice per

group). **f**, Experimental paradigm of mice treated with LPS and behavioral test (left) and immobility time in the FST. (n=6 mice per group). **g**, qRT-PCR analysis of FTO in the mPFC in the mice treated with LPS. (n=4 mice per group). **h**-**i**, Astrocytes were separated by flow cytometry (**h**), and the protein level of ALKBH5 detected by Simple western blot (**i**). (Ctrl, n=5; Sus, n=5; Res, n=4 mice). All data are presented as the mean ± SEM. Two-sided unpaired t-test (**f**, **g**) or One-way ANOVA with Bonferroni's multiple comparisons tests (**b**, **d**, **e**, **i**). * *p*< 0.05; ** *p*< 0.01; *** *p*< 0.001; n.s., no significance. See Supplementary Data 4 for statistical details

Supplementary Figure 2

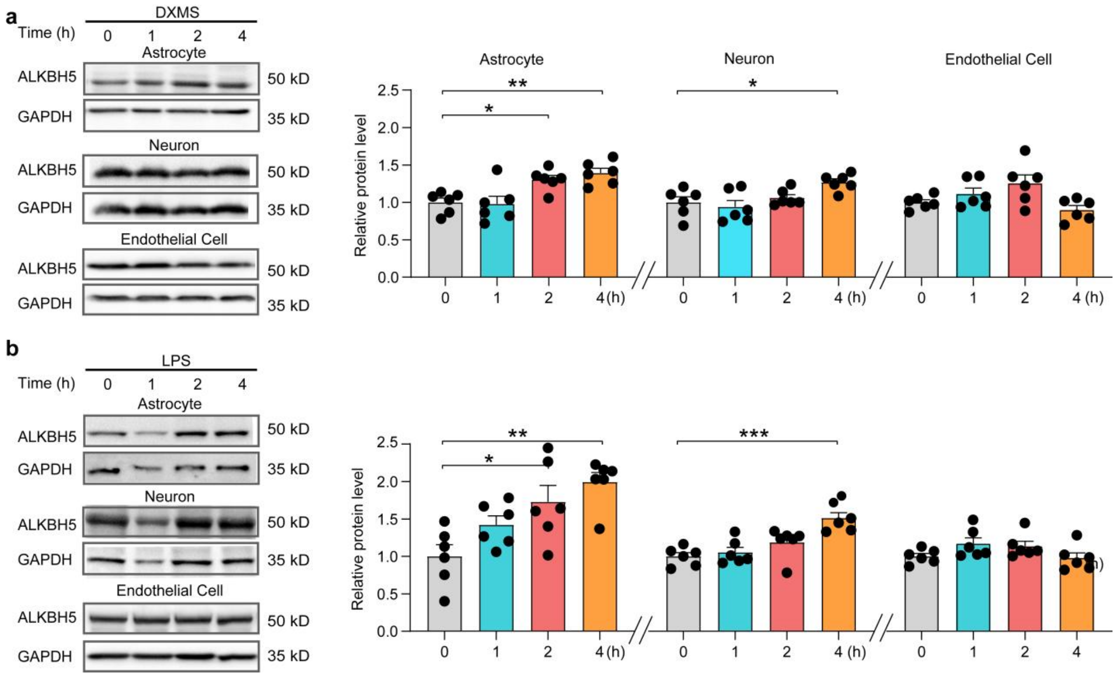

Supplementary Figure 2 The ALKBH5 expression in astrocytes, neurons and endothelial cells. a-b, Western blotting analysis of ALKBH5 protein in primary cultured astrocytes (DIV8), neurons (DIV14) and endothelial cells (bEnd3) treated with DXMS 1 μM (**a**), or LPS 1 μg mL^{-1} (**b**) for 0, 1, 2, 4 hours. (n=6 wells per group). All data are presented as the mean ± SEM. One-way ANOVA with Bonferroni's multiple comparisons test (**a** and **b**). * *p*< 0.05; ** *p*< 0.01; *** *p*< 0.001; n.s., no significance. See Supplementary Data 4 for statistical details

Supplementary Figure 3

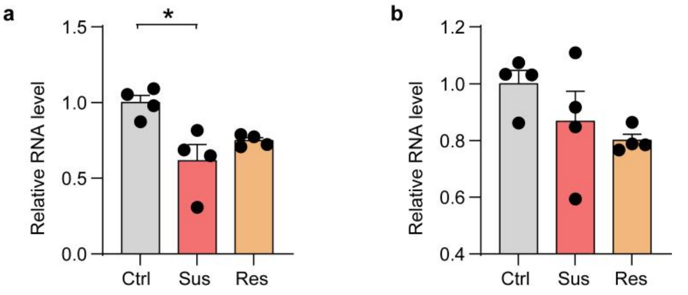

Supplementary Figure 3 The ALKBH5 expression in microglias and oligodendrocytes. a-b, qRT-PCR analysis of ALKBH5 in the mPFC of Sus, Res and Ctrl mice in microglias (**a**) and oligodendrocytes (**b**). (n=4 per group). Cells were separated by MACS. All data are presented as the mean ± SEM. One-way ANOVA with Bonferroni's multiple comparisons tests (**a** and **b**). * $p< 0.05$; ** $p< 0.01$; *** $p< 0.001$; n.s., no significance. See Supplementary Data 4 for statistical details

Supplementary Figure 4

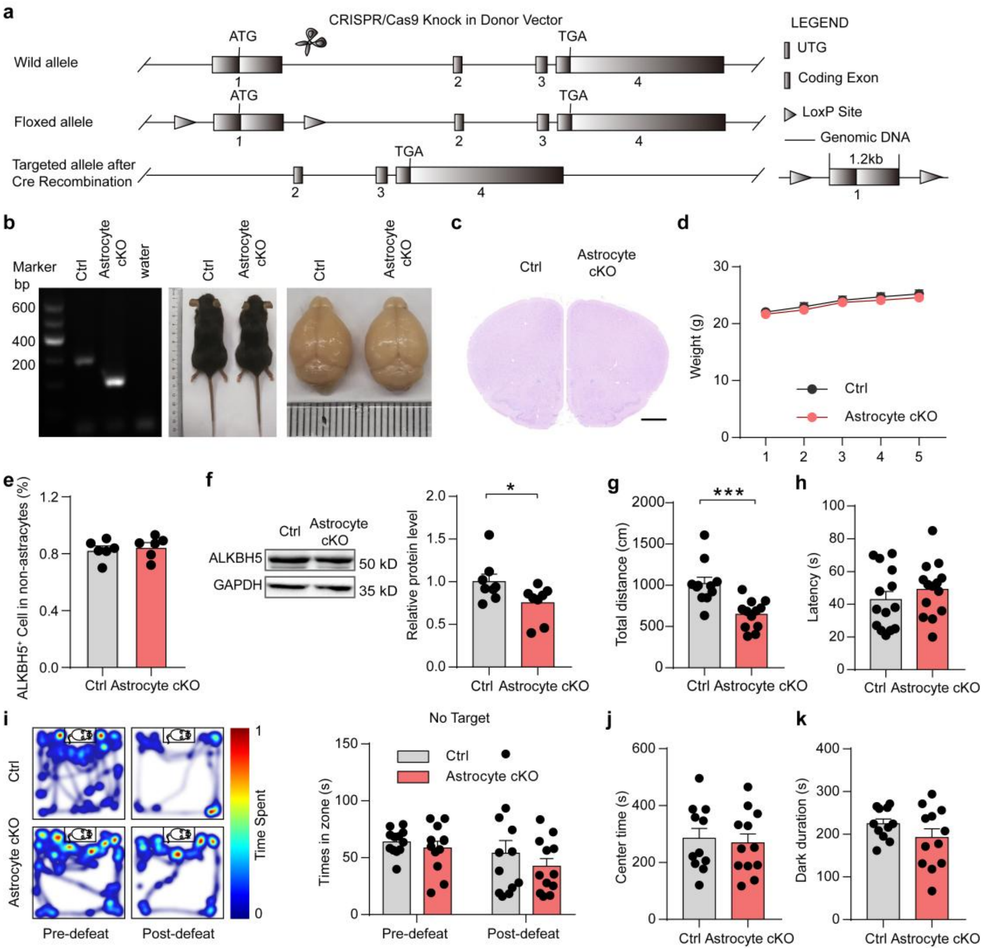

Supplementary Figure 4 Generation of *Fgfr3-iCreER^{T2}; Alkbh5$^{loxP/loxP}$* mice. a, Schematic of *Alkbh5$^{loxP/loxP}$* gene paradigm. **b**, Representative PCR genotyping, images and gross appearance of the brain of Astrocyte cKO and Ctrl mice. **c**, Representative images of H&E-stained coronal sections (Scale bars, 1000 μm). **d**, body weight of Astrocyte cKO and Ctrl mice (n=11 mice per group). **e**, Quantification of the ALKBH5 positive non-astrocytes of Astrocyte cKO and Ctrl mice. (n=6 mice per group). **f**, Western blotting analysis of the ALKBH5 in the mPFC of *Fgfr3-iCreER^{T2}; Alkbh5$^{loxP/loxP}$* (Astrocyte cKO) (n=8 mice per group) **g**, Total distance for Astrocyte cKO and Ctrl mice in the OFT. (**g**, Astrocyte cKO, n=12; Ctrl, n=11 mice). **h**, Latency to fall of Astrocyte cKO and Ctrl mice in the rota-rod test, n=14 mice per group. **i**, Time spent in the interaction zone before and after CSDS without target and heatmap of SI test. (n=12 mice per group). **j**, Center time for Astrocyte cKO and Ctrl mice in the open field test. (Astrocyte cKO, n=11; Ctrl, n=12 mice). **k**, Time spent in the dark box in the LD. (Astrocyte cKO, n=11; Ctrl, n=12 mice). All data are presented as the mean ± SEM. Two-sided unpaired t-test (**e**-**h**, **j**-**k**) or Two-way ANOVA with Bonferroni's multiple comparisons test (**d**, **i**). * $p< 0.05$; ** $p< 0.01$; *** $p< 0.001$, n.s., no significance. See Supplementary Data 4 for statistical details

Supplementary Figure 5

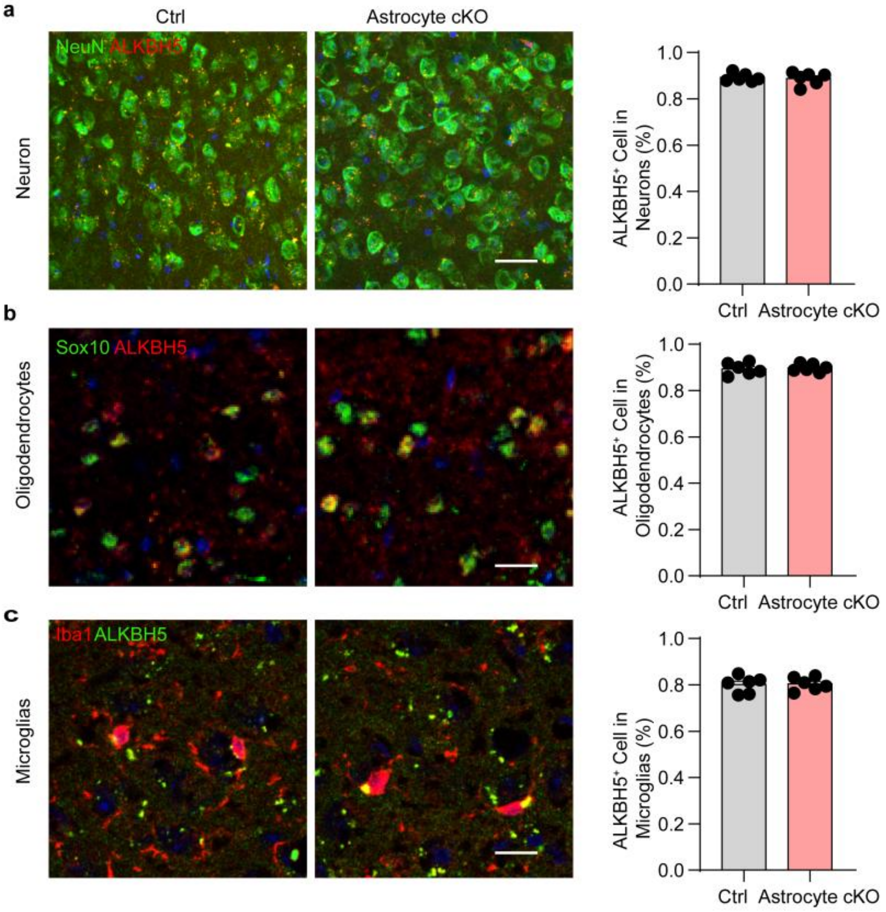

Supplementary Figure 5 Representative images and quantification of the ALKBH5 positive neurons, oligodendrocytes and microglias of Astrocyte cKO and Ctrl mice a, Representative images (red, ALKBH5; green, NeuN) (Left) and quantification of the ALKBH5 positive neurons (Right) of Astrocyte cKO and Ctrl mice, Scale bar=25 µm. (n=6 slice from 3 mice per group). **b**, Representative images (red, ALKBH5; green, Sox10) (Left) and quantification of the ALKBH5 positive oligodendrocytes (Right) of Astrocyte cKO and Ctrl mice, Scale bar=25 µm. (n=6 slice from 3 mice per group). **c**, Representative images (red, Iba1; green, ALKBH5) (Left) and quantification of the ALKBH5 positive microglias (Right) of Astrocyte cKO and Ctrl mice, Scale bar=20 µm. (n=6 slice from 3 mice per group). All data are presented as the mean ± SEM. Two-sided unpaired t-test (**a-c**). n.s., no significance. See Supplementary Data 4 for statistical details

Supplementary Figure 6

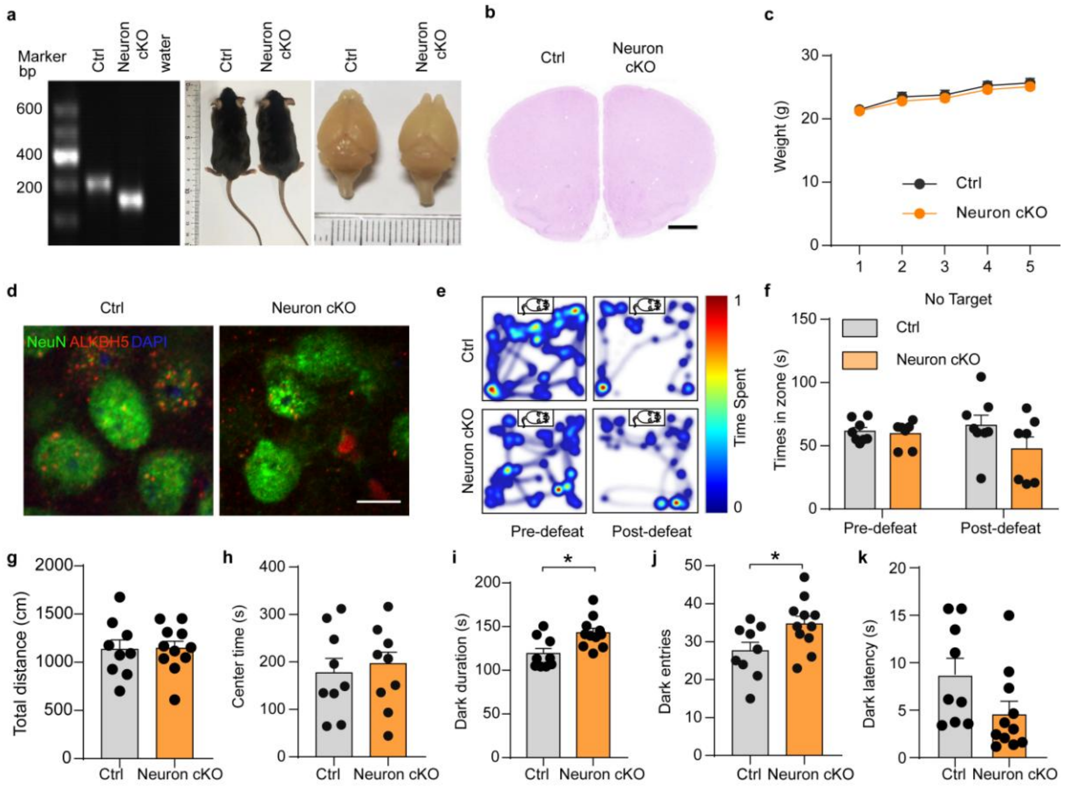

Supplementary Figure 6 Generation of *CaMK2a-iCreER^{T2}; Alkbh5$^{loxP/loxP}$* mice. a, Representative PCR genotyping, images and gross appearance of the brain of Neuron cKO and Ctrl mice. **b**, Representative images of H&E-stained coronal sections (Scale bars, 1000 μm). **c**, body weight of Neuron cKO and Ctrl mice (n=11 mice per group). **d**, Representative images and quantification of the ALKBH5 positive astrocytes (**c**, red, ALKBH5; green, NeuN) of cKO and Ctrl mice, Scale bar=20 μm. (n=4 mice per group). **e**, **f**, Time spent in the interaction zone before and after CSDS without target and heatmap of SI test. (Neuron cKO, n=7; Ctrl, n=8 mice). **g-h**, Total distance (**g**) and Center time (**h**) for Neuron cKO and Ctrl mice in the open field test. (Neuron cKO, n=11; Ctrl, n=9 mice). **i-k**, Time spent in the dark box (**i**), Dark latency (**k**),

Dark entries (**j**) in the dark box in the LD. (Neuron cKO, n=11; Ctrl, n=9 mice). All data are presented as the mean ± SEM. Two-sided unpaired t-test (**g-k**) or Two-way ANOVA with Bonferroni's multiple comparisons test (**c**, **f**). * $p< 0.05$; ** $p< 0.01$; *** $p< 0.001$, n.s., no significance. See Supplementary Data 4 for statistical details

Supplementary Figure 7

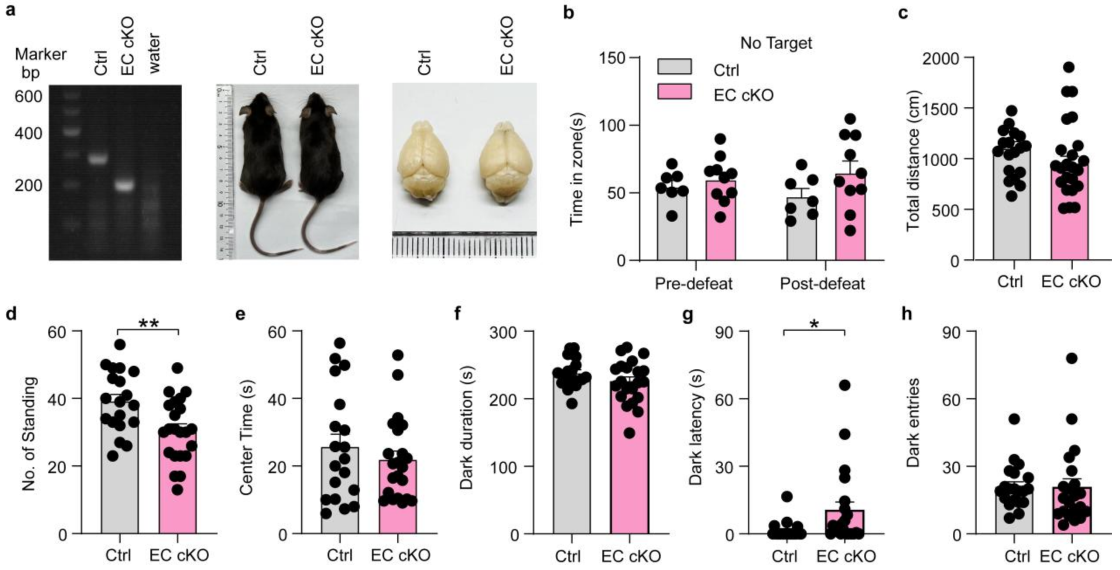

Supplementary Figure 7 Generation of *Cdh5-iCreER*^{T2}*; Alkbh5*$^{loxP/loxP}$ (EC cKO) mice. a, Representative PCR genotyping, images and gross appearance of the brain of EC cKO and Ctrl mice. **b**, Time spent in the interaction zone before and after CSDS without target and heatmap of SI test. (EC cKO, n=9; Ctrl, n=7 mice). **c-e**, Total distance (**c**), No. of standing (**d**), Center time (**e**) for EC cKO and Ctrl mice in the open field test. (EC cKO, n=22; Ctrl, n=19 mice). **f-h**, Time spent in the dark box (**f**), Dark latency (**g**), Dark entries (**h**) in the dark box in the LD. (EC cKO, n=21; Ctrl, n=20 mice). All data are presented as the mean ± SEM. Two-sided unpaired t-test (**c-h**) or Two-way ANOVA with Bonferroni's multiple comparisons test (**b**). * $p< 0.05$; ** $p< 0.01$; *** $p< 0.001$, n.s., no significance. See Supplementary Data 4 for statistical details

Supplementary Figure 8

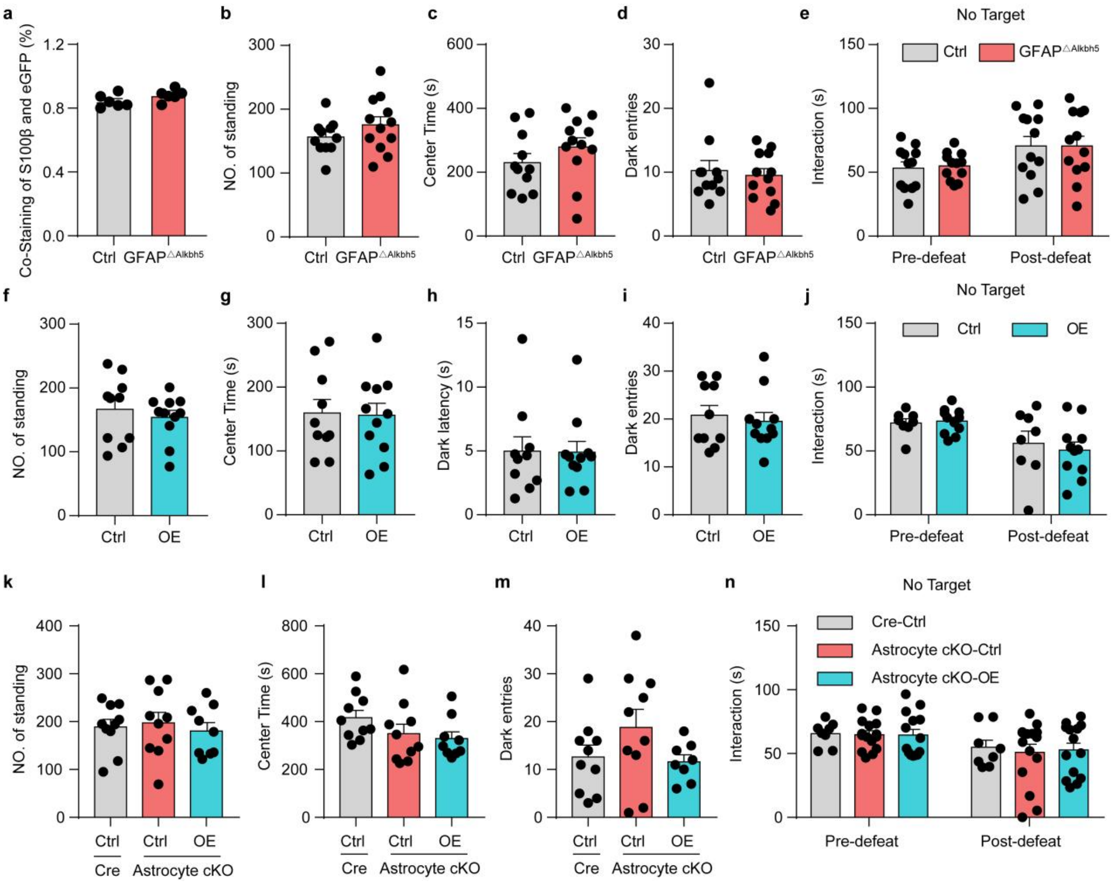

Supplementary Figure 8 Results of behavioral tests in the mice with Astrocyte-specific gain and loss of ALKBH5 in the mPFC. a, Quantification of the co-staining of S100β in GFAP positive cells in the mPFC of *Alkbh5*$^{loxP/loxP}$ mice. (n=6 mice per group). **b**, **f**, **k**, The number of standing in the open field test. (**b**, Ctrl, n=11; GFAP$^{\triangle Alkbh5}$ n=12 mice; **f**, Ctrl, n=10; OE, n=11 mice; **k**, Cre-Ctrl, n=10; cKO-Ctrl, n=10; cKO-OE, n=9 mice). **c**, **g**, **l**, Center time in the open field test. n=8-15 mice per group. **h**, Dark latency in the LD. (**c**, Ctrl, n=11; GFAP$^{\triangle Alkbh5}$ n=12 mice; **g**, Ctrl, n=10; OE, n=11 mice; **l**, Cre-Ctrl, n=10; cKO-Ctrl, n=10; cKO-OE, n=9 mice). **d**, **i**, **m**, Dark entries in the LD. (**d**, Ctrl, n=11; GFAP$^{\triangle Alkbh5}$ n=12 mice; **i**, Ctrl, n=10; OE, n=11 mice; **m**, Cre-Ctrl, n=10; cKO-Ctrl, n=10; cKO-OE, n=8 mice). **e**, **j**, **n**, Time spent in

the interaction zone before and after CSDS without target. (**b**, Ctrl, n=12; GFAP$^{\triangle Alkbh5}$ n=12 mice; **f**, Ctrl, n=8; OE, n=12 mice; **k**, Cre-Ctrl, n=8; cKO-Ctrl, n=15; cKO-OE, n=14 mice). All data are presented as the mean ± SEM. Two-sided unpaired t-test (**a-d**, **f-i**), One-way ANOVA (**k-m**) or Two-way ANOVA (**e**, **j** and **n**) with Bonferroni's multiple comparisons test. * $p< 0.05$; ** $p< 0.01$; *** $p< 0.001$, n.s., no significance. See Supplementary Data 4 for statistical details

Supplementary Figure 9

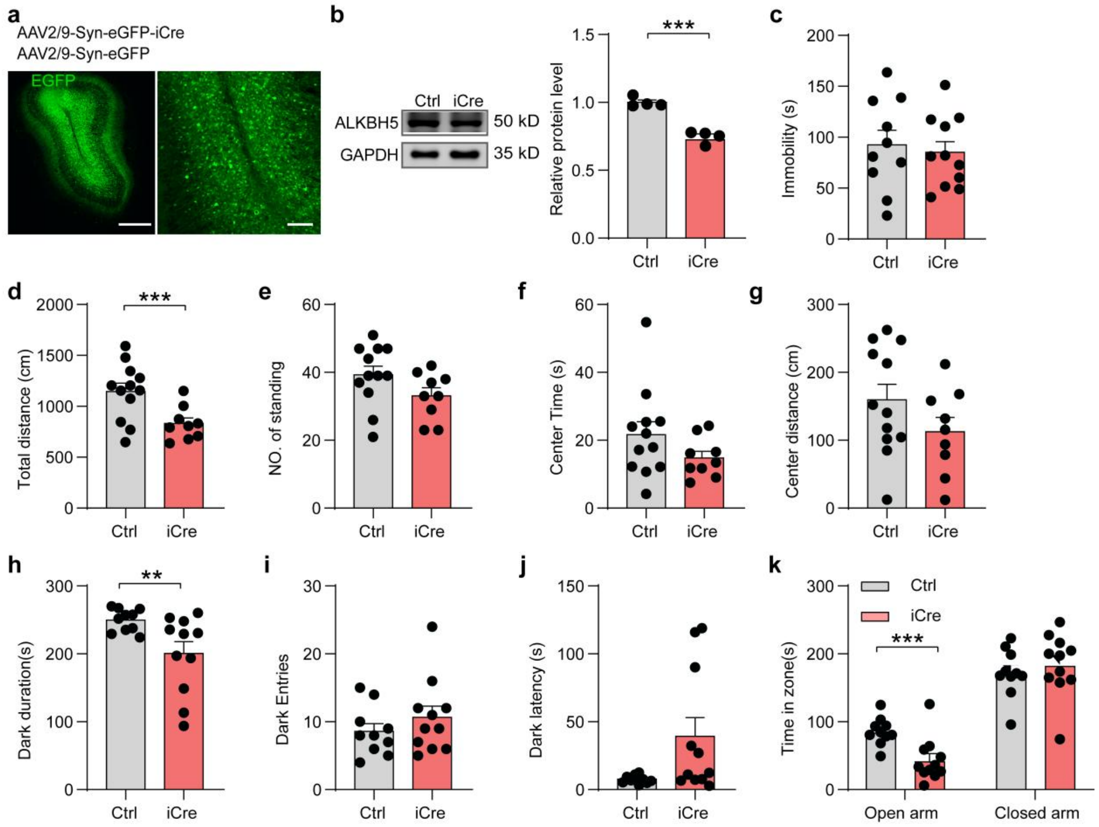

Supplementary Figure 9 Results of behavioral test of Neuron-specific knockout of ALKBH5 in olfactory bulb. **a**, Representative images of AAV-Syn-eGFP-iCre expression in the olfactory bulb of *Alkbh5$^{loxP/loxP}$* mice (iCre) (green, EGFP). Scale bars=500 μm (left), 25 μm (right). **b**, Western blotting analysis of ALKBH5 in the olfactory bulb of iCre and Ctrl mice. (n=4 mice per group). **c-k**, Statistics analysis of iCre and Ctrl mice in FST (**c**), OFT (**d-g**), LD (**h-j**) and EPM (**k**). (**c**, Ctrl, n=10; iCre n=11 mice; **d-g**, Ctrl, n=12; iCre n=9 mice; **h-k**, Ctrl, n=10; iCre n=11 mice). All data are presented as the mean ± SEM. Two-sided unpaired t-test (**b-k**). * $p< 0.05$; ** $p<$

0.01; *** $p< 0.001$, n.s., no significance. See Supplementary Data 4 for statistical details

Supplementary Figure 10

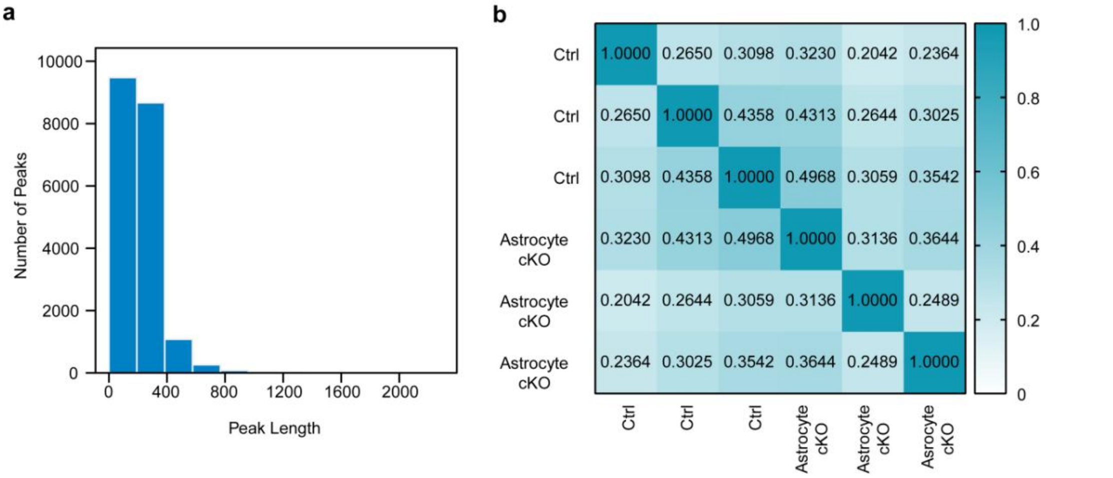

Supplementary Figure 10 The m6A Levels and distribution in Astrocytic ALKBH5 cKO mice. a, Peak length distribution map. **b**, Graph of the correlation coefficient between samples. See Supplementary Data 4 for statistical details

Supplementary Figure 11

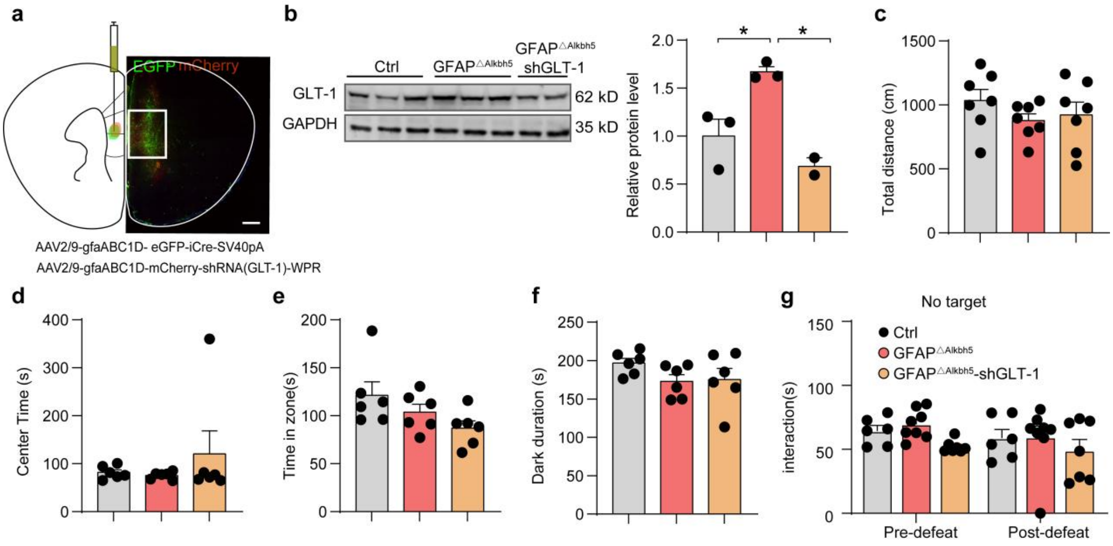

Supplementary Figure 11 Results of behavioral tests in the GFAP$^{\triangle Alkbh5}$ mice injected with GLT-1-shRNA in the mPFC. a, Representative images of AAV-gfaABC1D-mCherry-shRNA(GLT-1) expression in the mPFC of GFAP$^{\triangle Alkbh5}$

mice (green, GFAP; red, GLT-1). Scale bars=500 μm. **b**, Western blotting analysis of GLT-1 in the mPFC of GFAP$^{\triangle Alkbh5}$ mice. (Ctrl, n=3; GFAP$^{\triangle Alkbh5}$, n=3; GFAP$^{\triangle Alkbh5\text{-}shGLT\text{-}1}$, n=2 mice). **c**, Total distance in the open field test. (n=7 mice per group). **d**, Center time in the open field test. (n=6 mice per group). **e**, Time spent in the open arms in the EPM. (n=6 mice per group). **f**, Dark duration in the LD. (n=6 mice per group). **g**, Time spent in the interaction zone before and after CSDS without target. (Ctrl, n=6; GFAP$^{\triangle Alkbh5}$, n=8; GFAP$^{\triangle Alkbh5\text{-}shGLT\text{-}1}$, n=7 mice). All data are presented as the mean ± SEM. One-way ANOVA (**b-f**) or Two-way ANOVA (**g**) with Bonferroni's multiple comparisons test. * $p< 0.05$; ** $p< 0.01$; *** $p< 0.001$, n.s., no significance. See Supplementary Data 4 for statistical details

Supplementary Figure 12

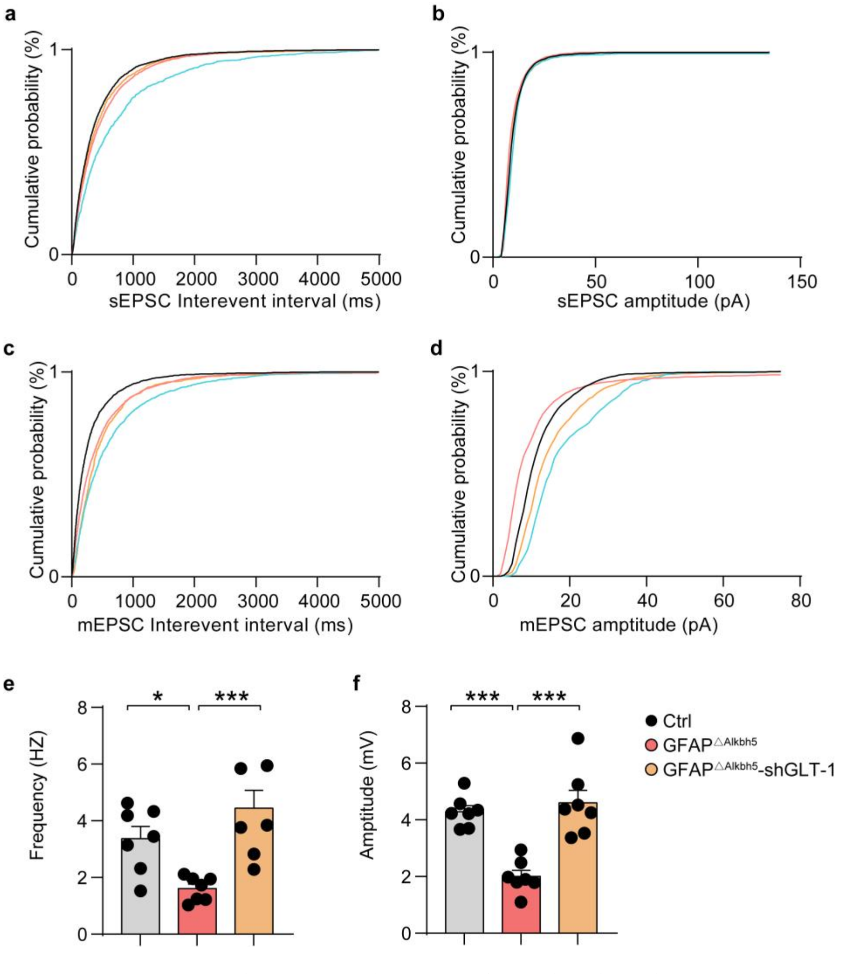

Supplementary Figure 12 Cumulative distribution curve of sEPSCs and mEPSC in Astrocyte ALKBH5 cKO mice. a-b, Cumulative distribution curve of sEPSCs

frequency (**a**) and amplitude (**b**). n=11-17 cells from 4 individual mice. **c**-**d**, Cumulative distribution curve of mEPSC frequency (**c**) and amplitudes (**d**). n=12-21 cells from 5 individual mice. **e-f**, Quantification of mEPSC frequency (**e**) and amplitudes (**f**). n=7 cells from 3 individual mice. All data are presented as the mean ± SEM. One-way ANOVA (**e-f**) with Bonferroni's multiple comparisons test. * $p< 0.05$; ** $p< 0.01$; *** $p< 0.001$; n.s., no significance. See Supplementary Data 4 for statistical details

Supplementary Figure 13

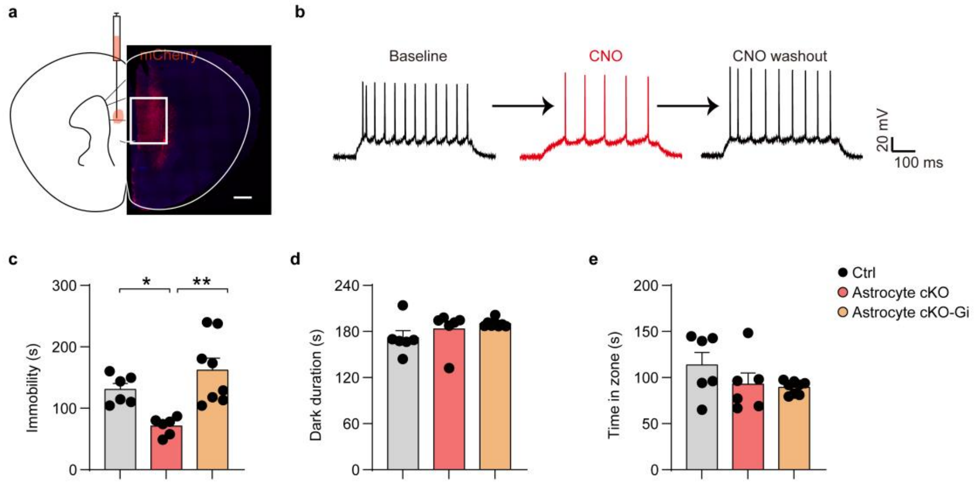

Supplementary Figure 13 Results of behavioral tests of inhibition of the activity of glutamatergic neurons. **a**, Representative images of CamkIIα-hM4D(Gi)-mCherry expression in the mPFC of Astrocyte cKO mice (red, mCherry). Scale bars=500 μm. **b**, Representative traces of whole-cell recording in an acute slice from mPFC neurons expressing hM4Di-mCherry after bath application of 1 μm CNO. **c**, Immobility time in the FST. (Ctrl, n=6; Astrocyte cKO, n=6; Astrocyte cKO-Gi, n=8 mice). **d**, Dark duration in the LD. (Ctrl, n=6; Astrocyte cKO, n=6; Astrocyte cKO-Gi, n=8 mice). **e**, Time spent in the open arms in the EPM. (Ctrl, n=6; Astrocyte cKO, n=6; Astrocyte cKO-Gi, n=8 mice). All data are presented the mean ± SEM. One-way ANOVA (**c-e**) with Bonferroni's multiple comparisons test. * $p< 0.05$; ** $p< 0.01$; *** $p< 0.001$; n.s., no significance. See Supplementary Data 4 for statistical details.

Supplementary Figure 14

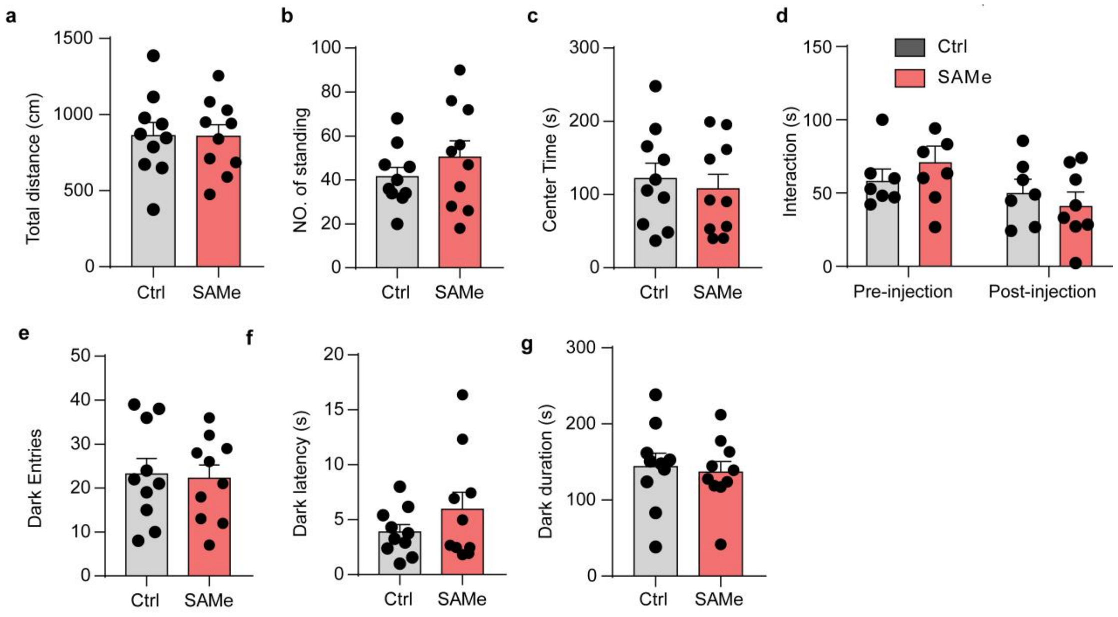

Supplementary Figure 14 Results of behavioral tests in the mice injected with SAMe. a-c, Total distance (**a**), the number of standing (**b**) and Center time (**c**) in the OFT. n=10 mice per group. **d**, Time spent in the interaction zone without target before and after injection SAMe. (Ctrl, n=7; SAMe, n=8 mice). **e-g**, Dark entries (**e**), dark latency (**f**) and Dark duration (**g**) in the LD. (n=10 mice per group). All data are presented as the mean ± SEM. Two-sided unpaired t-test (**a-c**, **e-g**) or two-way ANOVA (**d**) with Bonferroni's multiple comparisons test. * $p< 0.05$; ** $p< 0.01$; *** $p< 0.001$, n.s., no significance. See Supplementary Data 4 for statistical details
