# Supplementary material for: Astrocytic ALKBH5 in stress response contributes to depressive-like behaviors in mice
Source: Nat Commun. 2024 May 21;15:4347. doi: 10.1038/s41467-024-48730-2 (PMC11109195; doi:10.1038/s41467-024-48730-2)
Supplement: Supplementary file 3 — Description of Additional Supplementary Files [file 41467_2024_48730_MOESM3_ESM.pdf]

Description of Additional Supplementary Files

File name: Supplementary Data 1
Description: The human participants information.

File name: Supplementary Data 2
Description: Differentially expressed genes (DEGs) between Astrocyte cKO and Ctrl groups.

File name: Supplementary Data 3
Description: Differentially methylated peaks (DMPs) between Astrocyte cKO and Ctrl groups.

File name: Supplementary Data 4
Description: Statistical details of each figure.

File name: Supplementary Data 5
Description: The detailed information of the Sequences of oligonucleotides.
