# Supplementary material for: Astrocytic ALKBH5 in stress response contributes to depressive-like behaviors in mice
Source: Nat Commun. 2024 May 21;15:4347. doi: 10.1038/s41467-024-48730-2 (PMC11109195; doi:10.1038/s41467-024-48730-2)
Supplement: Supplementary file 8 — Supplementary Data 5 [file 41467_2024_48730_MOESM8_ESM.pdf]

Supplementary Data 5

Astrocytic ALKBH5 in stress response contributes to depressive-like behaviors in mice.

Supplementary table 1. The Sequences of qPCR primers in the human.

Gene name	Forward Primer	Reverse Primer
METTL3	TTCGAGAGGTGTCAGGGCT	CGGAAGGTTGGAGACAATGC
METTL14	CAGAGAACAAAGGAACACTGCC	TTGGTCCAACTGTGAGCCAG
WTAP	ACAACAGCAGGAGTCTGCAC	GATCTCAGTTGGGCAACGCT
FTO	GCACACGGGAAGGAGATGTT	ATGTCATCAGGATGGGCCAC
ALKBH5	CTATTCGGGTGTCGGAACCA	GGGTGCATCTAATCTTGTCTTCT
GAPDH	CGGAGTCAACGGATTTGGTC	TGGGTGGAATCATATTGGAACAT
YTHDF1	CATTATGAGAAGCGCCAGGA	AGATGCAACAATCAACCCCG
YTHDF2	ACCAACTCTAGGGACACTCA	GGATAAGGAGATGCAACCGT

Supplementary table 2. The Sequences of qPCR primers in the mice.

Gene name	Forward Primer	Reverse Primer
GAPDH	CAATGTGTCCGTCGTGGATCT	GTCCTCAGTGTAGCCCAAGATG
METTL3	ATTGAGAGACTGTCCCCTGG	AGCTTTGTAAGGAAGTGCGT
METTL14	AGACGCCTTCATCTCTTTGG	AGCCTCTCGATTTCCTCTGT
WTAP	GTTATGGCACGGGATGAGTT,	ATCTCCTGCTCTTTGGTTGC
FTO	CTGAGGAAGGAGTGGCATG	TCTCCACCTAAGACTTGTGC
ALKBH5	ACAAGATTAGATGCACCGCG	TGTCCATTTCCAGGATCCGG
YTHDF1	CATTATGAGAAGCGCCAGGA	AGATGCAACAATCAACCCCG
YTHDF2	ACCAACTCTAGGGACACTCA	GGATAAGGAGATGCAACCGT
YTHDF3	TGCACATTATGAAAAGCGTCA	AGATGCGCTGATGAAAACCA)
YTHDC1	TTCATAACATGGGACCACCG	TCATAGTCATGTACTCGTTTATCTC
Hnrnpc	CAAACGTCAGCGTGTTTCAG	TGGGGATGAGAAGGACAAGT
Hnrnpa2B1	GTGGAGGGAACTATGGTCCT	TGAAGGCACCAACAAGAACT

Supplementary table 3. The Sequences of SLCLA2-3' UTR with wild-type m6A sites in the Luciferase assay of Fig.5b.

TTCATGGTCACCGCATCTACAGTAGTCTCTGGTTAAACCCAAAAGAAGGATTACTGAGT
GCCAACCCAGCCAGGTGGCAACTGTGACAGCTAATTTTCCGTGGGTGGAGTGAGATAA
AGTCTCATCAAGAACACCAACCAGCTTAGGATTTTCCCTAAAACTCTCTTCCCAGTTCA
CTGCCCTCGCCCTTCCCTTTCCTTTCGTTTCATTGCTTAAGAG**A**CTGCTTAGTAAGTGGC
CAAATAGTTAAGAGGGGGTTGGCTTGGAAGCAGATAACAGGAGATCTTAACTATCAGT
AATAGCCAGTCTCACCGCCTCACACCATAATCAGGCAATGACACAGAGATGTTTAAAA
ACAACGTGGCTTGGGAGAGGAAACATTTGATTAAGACACTCCCCATCTGTAGCTGTAA
GGCTCACACAGCCACTGTGTGCCCAGGTTTCTTGCACTTCCTT

Supplementary table 4. The Sequences of sgRNA primers for single-base editing in the Fig.5c.

sgRNA	Forward Primer	Reverse Primer
SPACER-M	ccggttaagagactgcttagtaag	aaaccttactaagcagtctcttaa

Supplementary table 5. The Sequences of GLT-1-specific m6A qPCR primers in the Fig.7g.

Gene name	Forward Primer	Reverse Primer
GLT-1	ATTTTCCGTGGGTGGAGTGAG	CCAAGCCAACCCCCTCTTAAC
