# Supplementary material for: Astrocytic ALKBH5 in stress response contributes to depressive-like behaviors in mice
Source: Nat Commun. 2024 May 21;15:4347. doi: 10.1038/s41467-024-48730-2 (PMC11109195; doi:10.1038/s41467-024-48730-2)
Supplement: Supplementary file 9 — Reporting Summary [file 41467_2024_48730_MOESM9_ESM.pdf]

nature portfolio

Corresponding author(s): Xiong Cao

Last updated by author(s): Apr 1, 2024

Reporting Summary

Nature Portfolio wishes to improve the reproducibility of the work that we publish. This form provides structure for consistency and transparency in reporting. For further information on Nature Portfolio policies, see our Editorial Policies and the Editorial Policy Checklist.

Statistics

For all statistical analyses, confirm that the following items are present in the figure legend, table legend, main text, or Methods section.

n/a	Confirmed	
☐	☒	The exact sample size (*n*) for each experimental group/condition, given as a discrete number and unit of measurement
☐	☒	A statement on whether measurements were taken from distinct samples or whether the same sample was measured repeatedly
☐	☒	The statistical test(s) used AND whether they are one- or two-sided<br>*Only common tests should be described solely by name; describe more complex techniques in the Methods section.*
☐	☒	A description of all covariates tested
☐	☒	A description of any assumptions or corrections, such as tests of normality and adjustment for multiple comparisons
☐	☒	A full description of the statistical parameters including central tendency (e.g. means) or other basic estimates (e.g. regression coefficient) AND variation (e.g. standard deviation) or associated estimates of uncertainty (e.g. confidence intervals)
☐	☒	For null hypothesis testing, the test statistic (e.g. *F*, *t*, *r*) with confidence intervals, effect sizes, degrees of freedom and *P* value noted<br>*Give P values as exact values whenever suitable.*
☐	☒	For Bayesian analysis, information on the choice of priors and Markov chain Monte Carlo settings
☐	☒	For hierarchical and complex designs, identification of the appropriate level for tests and full reporting of outcomes
☒	☐	Estimates of effect sizes (e.g. Cohen's *d*, Pearson's *r*), indicating how they were calculated

Our web collection on statistics for biologists contains articles on many of the points above.

Software and code

Policy information about availability of computer code

Data collection

For slice physiological recording, the current and voltage signals were recorded with HEKA EPC10 and Patch Master software (HEKA, Germany). EthoVision video tracking system (Noldus, the Netherlands) was used for forced swimming test, elevated plus maze test an social interaction test. For open field test and light/dark test, VersaMax (VersaMax Animal Activity Monitor system, the USA) was used. The rotarod was recorded by AccuRotor Rota Rod (AccuScan Instruments Inc.; Columbus, OH). Images were captured on a NikonA1R (Nikon, Japan). Flowcytometry data were collected using BD LSRFortessa X-20 (BD, USA). The Simple Western system were used to collect protein data (Protein Simple, Bio-Techne, USA). Western blot bands were obtained by using Image lab (Bio- rad, the USA) software.

Data analysis

For slice physiological recording, the data was analyzed using Patch Master software (HEKA, Germany). Behavioral videos analyzed using EthoVision XT 11 software (Noldus) and VersaMax 4.20 software.. Flowcytometry data were analyzed using FlowJo V10. SPSS 20.0 (IBM SPSS, USA) were used for the statistical analyses. Imaging data were processed and analyzed using Imaris 8.0 (Oxford Instruments, the USA) or NIS-Elements Viewer (Nikon, Japan). Image J(1.4.3.67) was used for cell count and was used for spine analysis. Image lab (Bio-rad, the USA) was used for western blot analysis. For FIT TEST, Bulk fluorescence signals were acquired and analyzed with MATLAB software. The z-score for a population of astrocytes was calculated using the following formula: z-score=(FSignal – FBasal)/STD (FBasal).
For DEGs analysis, the limma Bioconductor package was then used to analyze the gene expression data.
The quantity and the integrity of RNA yield were assessed by using the K5500 (Beijing Kaiao, China) and the Agilent 2200 TapeStation (Agilent Technologies, USA), respectively. m6A antibody immunoprecipitation RNA was quality control with Qubit (Thermo Fisher Scientifc, USA) and Agilent 2200 TapeStation (Agilent Technologies, USA). The final library product was assessed with Agilent 2200 TapeStation and Qubit® (Life Technologies, USA) and then sequenced on Illumina (Illumina, USA) platformat with pair-end 150 bp at Ribobio Co. Ltd (Ribobio, China).
Adaptor and low-quality bases were trimmed with Trimmomatictools(version:0.36), and the clean reads underwent rRNA deleting through RNAcentral to get effective reads. Genomic alignment (version from UCSC genome browser) was using Tophat(version:2.0.13) to get uniquely mapping reads.

Effective reads from the input samples was used for RNA-seq analysis, and the reads count value of each transcript was calculated by HTSeq(version:0.6.0). Differential expression genes were identified by the DESeq2 R package.
MACS2(version 2.1.0.20151222) was employed to perform m6A peak calling. Then Homer(version:4.8) was used to annotate the m6A Peaks. The nucleotides in the m6A Peaks region were used for detection of the consensus m6A motif by DREME (version:4.11.1) and MEME(version:4.11.1). Motif central enrichment was performed by CentriMo (version:4.11.1). Differential methylation was determined by MetDiff
Gene ontology (GO) and Kyoto Encyclopedia of Genes and Genomes (KEGG) pathway enrichment analysis were performed using ClusterProfiler R package/ KOBAS3.0. The results from the enrichment analysis were restricted to GO biological process and KEGG pathway terms, with adjusted P-value <0.05 considered to be significant.

For manuscripts utilizing custom algorithms or software that are central to the research but not yet described in published literature, software must be made available to editors and reviewers. We strongly encourage code deposition in a community repository (e.g. GitHub). See the Nature Portfolio guidelines for submitting code & software for further information.

Data

Policy information about availability of data

All manuscripts must include a data availability statement. This statement should provide the following information, where applicable:

- Accession codes, unique identifiers, or web links for publicly available datasets
- A description of any restrictions on data availability
- For clinical datasets or third party data, please ensure that the statement adheres to our policy

The MeRIP-seq data generated in this study have been deposited in the Genome Sequence Archive under accession code CRA012006 (https://ngdc.cncb.ac.cn/gsa). Previously described data was downloaded from the GEO database (GSE102556) (https://www.ncbi.nlm.nih.gov/geo/query/acc.cgi?acc=GSE102556). Uncropped and unprocessed scans are supplied in the Source data. Source data are provided as a Source Data.xlsx. Source data are provided with this paper.

Research involving human participants, their data, or biological material

Policy information about studies with human participants or human data. See also policy information about sex, gender (identity/presentation), and sexual orientation and race, ethnicity and racism.

Reporting on sex and gender	No sex- and gender- based analysis have been performed because of the small sample size. We performed quantitative PCR analysis of peripheral blood of 30 MDD patients (19 females and 11 males) aged 24.4 ± 6.61 (SD) years and healthy controls (20 females and 18 males) aged 25.08 ± 2.53 (SD) years. Sex and gender were considered in the study design. The sex data are provided in the Supplementary Information. The MDD and healthy controls were recruited from the Department of Psychology and Behavior, Guangdong 999 Brain Hospital, Guangzhou, China.
Reporting on race, ethnicity, or other socially relevant groupings	No race- and ethnicity- based analysis have been performed. None of these patients with MDD or healthy controls had taken any psychotropic medications within 4 weeks. All of the patients were clinically diagnosed by at least two psychiatrists in accordance with the Diagnostic and Statistical Manual of Mental Disorders Fourth Edition (DSM-IV) criteria and the 17-item Hamilton Rating Scale for Depression (HAMD-17).
Population characteristics	We performed quantitative PCR analysis of peripheral blood of 30 MDD patients (19 females and 11 males) aged 24.4 ± 6.61 (SD) years and healthy controls (20 females and 18 males) aged 25.08 ± 2.53 (SD) years. No Sex- and gender- based analysis have been performed. The sex data are provided in the Supplementary Information.
Recruitment	Participants were recruited from Guangdong 999 Brain Hospital, China. None of patients with MDD or healthy controls had taken any psychotropic medcations within 4 weeks. The participants have signed an informed consent form and have not been offered any compensation.
Ethics oversight	The study with the use of peripheral blood from the MDD patients was approved by the Ethics Committee of Guangdong 999 Brain Hospital, Guangzhou (NO. 2021-01-087). Research was performed in accordance to the Declaration of Helsinki (ethical principles for medical research involving human subjects).

Note that full information on the approval of the study protocol must also be provided in the manuscript.

Field-specific reporting

Please select the one below that is the best fit for your research. If you are not sure, read the appropriate sections before making your selection.

☒ Life sciences ☐ Behavioural & social sciences ☐ Ecological, evolutionary & environmental sciences

For a reference copy of the document with all sections, see nature.com/documents/nr-reporting-summary-flat.pdf

Life sciences study design

All studies must disclose on these points even when the disclosure is negative.

Sample size	No statistical methods were used to pre-determine sample sizes but our sample sizes are similar to those reported in previous publications (PMID: 31651582; PMID: 34233151; PMID: 36443522).

Data exclusions	For all experiments, mice with signs of infection/bleeding/unhealthy conditions after the surgeries were excluded for behavioral tests and mice with missed viral injections or implantation targets, as described by brain atlas, were not included in experimental analyses.
Replication	Behavioral experiments are replicated multiples times with independent mice, and at least two people independently analyzed time points for the behavioral events. Measurements are taken from individual mice/cells from multiple biological replicates. All these are described in Methods and in the results. Numbers of replicates (n) are indicated in the figure legends.
Randomization	The animals in the experiments were randomized assigned. Cells cultures were randomized allocated into experimental groups. For human participants, all of the patients were clinically diagnosed by at least two psychiatrists in accordance with the Diagnostic and Statistical Manual of Mental Disorders Fourth Edition (DSM-IV) criteria and the 17-item Hamilton Rating Scale for Depression (HAMD-17). It employs a case-matched control comparisons in which samples of the Ctrl and MDD groups with age- and sex-matched.
Blinding	All investigators were blinded to group allocation during data collection and analysis.

Behavioural & social sciences study design

All studies must disclose on these points even when the disclosure is negative.

Study description	*Briefly describe the study type including whether data are quantitative, qualitative, or mixed-methods (e.g. qualitative cross-sectional, quantitative experimental, mixed-methods case study).*
Research sample	*State the research sample (e.g. Harvard university undergraduates, villagers in rural India) and provide relevant demographic information (e.g. age, sex) and indicate whether the sample is representative. Provide a rationale for the study sample chosen. For studies involving existing datasets, please describe the dataset and source.*
Sampling strategy	*Describe the sampling procedure (e.g. random, snowball, stratified, convenience). Describe the statistical methods that were used to predetermine sample size OR if no sample-size calculation was performed, describe how sample sizes were chosen and provide a rationale for why these sample sizes are sufficient. For qualitative data, please indicate whether data saturation was considered, and what criteria were used to decide that no further sampling was needed.*
Data collection	*Provide details about the data collection procedure, including the instruments or devices used to record the data (e.g. pen and paper, computer, eye tracker, video or audio equipment) whether anyone was present besides the participant(s) and the researcher, and whether the researcher was blind to experimental condition and/or the study hypothesis during data collection.*
Timing	*Indicate the start and stop dates of data collection. If there is a gap between collection periods, state the dates for each sample cohort.*
Data exclusions	*If no data were excluded from the analyses, state so OR if data were excluded, provide the exact number of exclusions and the rationale behind them, indicating whether exclusion criteria were pre-established.*
Non-participation	*State how many participants dropped out/declined participation and the reason(s) given OR provide response rate OR state that no participants dropped out/declined participation.*
Randomization	*If participants were not allocated into experimental groups, state so OR describe how participants were allocated to groups, and if allocation was not random, describe how covariates were controlled.*

Ecological, evolutionary & environmental sciences study design

All studies must disclose on these points even when the disclosure is negative.

Study description	*Briefly describe the study. For quantitative data include treatment factors and interactions, design structure (e.g. factorial, nested, hierarchical), nature and number of experimental units and replicates.*
Research sample	*Describe the research sample (e.g. a group of tagged Passer domesticus, all Stenocereus thurberi within Organ Pipe Cactus National Monument), and provide a rationale for the sample choice. When relevant, describe the organism taxa, source, sex, age range and any manipulations. State what population the sample is meant to represent when applicable. For studies involving existing datasets, describe the data and its source.*
Sampling strategy	*Note the sampling procedure. Describe the statistical methods that were used to predetermine sample size OR if no sample-size calculation was performed, describe how sample sizes were chosen and provide a rationale for why these sample sizes are sufficient.*
Data collection	*Describe the data collection procedure, including who recorded the data and how.*
Timing and spatial scale	*Indicate the start and stop dates of data collection, noting the frequency and periodicity of sampling and providing a rationale for these choices. If there is a gap between collection periods, state the dates for each sample cohort. Specify the spatial scale from which the data are taken*
Data exclusions	*If no data were excluded from the analyses, state so OR if data were excluded, describe the exclusions and the rationale behind them, indicating whether exclusion criteria were pre-established.*

Reproducibility	*Describe the measures taken to verify the reproducibility of experimental findings. For each experiment, note whether any attempts to repeat the experiment failed OR state that all attempts to repeat the experiment were successful.*
Randomization	*Describe how samples/organisms/participants were allocated into groups. If allocation was not random, describe how covariates were controlled. If this is not relevant to your study, explain why.*
Blinding	*Describe the extent of blinding used during data acquisition and analysis. If blinding was not possible, describe why OR explain why blinding was not relevant to your study.*

Did the study involve field work? ☐ Yes ☐ No

Field work, collection and transport

Field conditions	*Describe the study conditions for field work, providing relevant parameters (e.g. temperature, rainfall).*
Location	*State the location of the sampling or experiment, providing relevant parameters (e.g. latitude and longitude, elevation, water depth).*
Access & import/export	*Describe the efforts you have made to access habitats and to collect and import/export your samples in a responsible manner and in compliance with local, national and international laws, noting any permits that were obtained (give the name of the issuing authority, the date of issue, and any identifying information).*
Disturbance	*Describe any disturbance caused by the study and how it was minimized.*

Reporting for specific materials, systems and methods

We require information from authors about some types of materials, experimental systems and methods used in many studies. Here, indicate whether each material, system or method listed is relevant to your study. If you are not sure if a list item applies to your research, read the appropriate section before selecting a response.

Materials & experimental systems

n/a	Involved in the study	
☐	☒	Antibodies
☒	☐	Eukaryotic cell lines
☒	☐	Palaeontology and archaeology
☐	☒	Animals and other organisms
☒	☐	Clinical data
☒	☐	Dual use research of concern
☒	☐	Plants

Methods

n/a	Involved in the study	
☒	☐	ChIP-seq
☐	☒	Flow cytometry
☒	☐	MRI-based neuroimaging

Antibodies

Antibodies used	For Western blot assays, anti-ALKBH5 (Sigma: HPA007196, 1:1000), anti-GLT-1 (Abcam: Ab205247, 1:1000), anti-GAPDH (Proteintech: 60004, 1:5000), HRP Goat Anti-Mouse IgG (H+L) (Abclonal, AS003, 1:5000), HRP Goat Anti-Rabbit IgG (H+L) (Abclonal, AS014, 1:5000).<br>For immunohistochemistry, anti-ALKBH5 (Sigma: HPA007196, 1:300), anti-NeuN (Millipore: MAB377, 1:300), anti-GFAP (CST:3670, 1:300). anti-Iba1 (Abcam: ab178846, 1:500), anti-Sox10 (Abcam: ab155279, 1:500), anti-S100β (Abcam: ab52642, 1:300), Alexa fluor 488-anti-Mouse secondary antibody (Invitrogen, A32723, 1:500), Alexa fluor 568-anti-Mouse secondary antibody (Invitrogen, A11004, 1:500), Alexa fluor 488-anti-Rabbit secondary antibody (Invitrogen, A32731, 1:500), Alexa fluor 568-anti-Rabbit secondary antibody (Invitrogen, A11036, 1:500).<br>For isolation of brain astrocytes and flow cytometry, ACSA-2-PE of the FACS antibodies (Miltenyi Biotec, 130-102-365, Germany).<br>For magnetic-activated cell sorting, anti-CD11b immunomagnetic beads (Miltenyi Biotec: 130-093-634, Germany), anti-oligodendrocytes immunomagnetic beads (Miltenyi Biotec: 130-094-534, Germany).
Validation	ALKBH5: extensively validated in the literature, in mouse and human tissue: https://www.sigmaaldrich.cn/CN/zh/product/sigma/hpa007196.<br>GLT-1, Abcam, https://www.abcam.cn/products/primary-antibodies/eaat2-antibody-epr19794- ab205247.html, validated in multiple publications in mouse and human tissue.<br>Iba1, Abcam, https://www.abcam.cn/products/primary-antibodies/iba1-antibody-epr16588-ab178846.html, validated in multiple publications in mouse, rat, human tissue.<br>Sox10, Abcam, https://www.abcam.cn/products/primary-antibodies/sox10-antibody-epr4007-ab155279.html, validated in multiple publications in mouse, rat, human tissue.<br>S100β, Abcam, https://www.abcam.cn/products/primary-antibodies/s100-beta-antibody-ep1576y-astrocyte-marker-ab52642.html, validated in multiple publications in mouse, rat, human tissue.<br>GAPDH, Proteintech, https://www.ptgcn.com/products/GAPDH-Antibody-60004-1-Ig.htm, validated in multiple publications in

mouse, rat, human tissue.
NeuN, Millipore, https://www.sigmaaldrich.cn/CN/zh/product/mm/mab377, validated in multiple publications in mouse, rat, human tissue.
GFAP, CST, https://www.cellsignal.cn/products/primary-antibodies/gfap-ga5-mouse-mab/3670, validated in multiple publications in mouse, rat, human tissue.
The secondary antibody for IHC was purchased in Invitrogen and all of them were validated in multiple publications in mouse, rat, human tissue. The manufacturer's websites are listed as follows:
Alexa fluor 488-anti-Mouse: https://www.thermofisher.cn/cn/zh/antibody/product/Goat-anti-Mouse-IgG-H-L-Highly-Cross-Adsorbed-Secondary-Antibody-Polyclonal/A32723.
Alexa fluor 488-anti-Rabbit: https://www.thermofisher.cn/cn/zh/antibody/product/Goat-anti-Rabbit-IgG-H-L-Highly-Cross-Adsorbed-Secondary-Antibody-Polyclonal/A32731.
Alexa fluor 568-anti-Mouse: https://www.thermofisher.cn/cn/zh/antibody/product/Goat-anti-Mouse-IgG-H-L-Cross-Adsorbed-Secondary-Antibody-Polyclonal/A-11004.
Alexa fluor 568-anti-Rabbit: https://www.thermofisher.cn/cn/zh/antibody/product/Goat-anti-Rabbit-IgG-H-L-Highly-Cross-Adsorbed-Secondary-Antibody-Polyclonal/A-11036
The secondary antibody for WB was purchased in Abclone and all of them were validated in multiple publications in mouse, rat, human tissue. The manufacturer's websites are listed as follows:
HRP Goat Anti-Mouse lgG (H+L): https://abclonal.com.cn/catalog/AS003.
HRP Goat Anti-Rabbit lgG (H+L): https://abclonal.com.cn/catalog/AS014.
For isolation of flow cytometry or magnetic-activated cell sorting, the antibody was purchased in Miltenyi and all of them were validated in multiple publications in mouse brains. The manufacturer's websites are listed as follows:
ACSA-2-PE of the FACS antibodies: https://www.miltenyibiotec.com/CN-en/products/acsa-2-antibody-anti-mouse-ih3-18a3.html#conjugate=pe:size=30-ug-in-200-ul.
anti-CD11b immunomagnetic beads: https://www.miltenyibiotec.com/CN-en/products/cd11b-microglia-microbeads-human-and-mouse.html#size=for-1%C3%9710-sup-9-sup-total-cells.
anti-oligodendrocytes immunomagnetic beads: https://www.miltenyibiotec.com/CN-en/products/anti-o4-microbeads-human-mouse-rat.html#size=for-1%C3%9710-sup-9-sup-total-cells.

Eukaryotic cell lines

Policy information about cell lines and Sex and Gender in Research

Cell line source(s)
State the source of each cell line used and the sex of all primary cell lines and cells derived from human participants or vertebrate models.

Authentication
Describe the authentication procedures for each cell line used OR declare that none of the cell lines used were authenticated.

Mycoplasma contamination
Confirm that all cell lines tested negative for mycoplasma contamination OR describe the results of the testing for mycoplasma contamination OR declare that the cell lines were not tested for mycoplasma contamination.

Commonly misidentified lines (See ICLAC register)
Name any commonly misidentified cell lines used in the study and provide a rationale for their use.

Palaeontology and Archaeology

Specimen provenance
Provide provenance information for specimens and describe permits that were obtained for the work (including the name of the issuing authority, the date of issue, and any identifying information). Permits should encompass collection and, where applicable, export.

Specimen deposition
Indicate where the specimens have been deposited to permit free access by other researchers.

Dating methods
If new dates are provided, describe how they were obtained (e.g. collection, storage, sample pretreatment and measurement), where they were obtained (i.e. lab name), the calibration program and the protocol for quality assurance OR state that no new dates are provided.

☐ Tick this box to confirm that the raw and calibrated dates are available in the paper or in Supplementary Information.

Ethics oversight
Identify the organization(s) that approved or provided guidance on the study protocol, OR state that no ethical approval or guidance was required and explain why not.

Note that full information on the approval of the study protocol must also be provided in the manuscript.

Animals and other research organisms

Policy information about studies involving animals; ARRIVE guidelines recommended for reporting animal research, and Sex and Gender in Research

Laboratory animals
C57BL/6J mice aged 8-10 weeks purchased from Charles River used in this study; Alkbh5loxP/loxP mice were purchased from Shanghai Model Organisms Center (No. NM-CKO-190004); CaMKⅡα-creERT2 mice were purchased from Jackson Laboratories (Stock No. 012362). Fgfr3-iCreERT2 mice (C57BL/6J background) were generously provided by William D Richardson (University

	College London). Cdh5CreERT2 mice were purchased from Stock No. 004860 (Stock No. 110140). The adult male CD1 mice (CD-1®(ICR) IGS) (older than 5 months of age) were purchased from Charles River Laboratories (Beijing).
Wild animals	The study did not involve wild animals.
Reporting on sex	Male and female mice were used in all experiments.
Field-collected samples	This study did not involve samples collected from the field.
Ethics oversight	All of the experiments were conducted in accordance with the Regulations for the Administration of Affairs Concerning Experimental Animals (China) and were approved by the Southern Medical University Animal Ethics Committee.

Note that full information on the approval of the study protocol must also be provided in the manuscript.

Clinical data

Policy information about clinical studies

All manuscripts should comply with the ICMJE guidelines for publication of clinical research and a completed CONSORT checklist must be included with all submissions.

Clinical trial registration	*Provide the trial registration number from ClinicalTrials.gov or an equivalent agency.*
Study protocol	*Note where the full trial protocol can be accessed OR if not available, explain why.*
Data collection	*Describe the settings and locales of data collection, noting the time periods of recruitment and data collection.*
Outcomes	*Describe how you pre-defined primary and secondary outcome measures and how you assessed these measures.*

Dual use research of concern

Policy information about dual use research of concern

Hazards

Could the accidental, deliberate or reckless misuse of agents or technologies generated in the work, or the application of information presented in the manuscript, pose a threat to:

No	Yes	
☐	☐	Public health
☐	☐	National security
☐	☐	Crops and/or livestock
☐	☐	Ecosystems
☐	☐	Any other significant area

Experiments of concern

Does the work involve any of these experiments of concern:

No	Yes	
☐	☐	Demonstrate how to render a vaccine ineffective
☐	☐	Confer resistance to therapeutically useful antibiotics or antiviral agents
☐	☐	Enhance the virulence of a pathogen or render a nonpathogen virulent
☐	☐	Increase transmissibility of a pathogen
☐	☐	Alter the host range of a pathogen
☐	☐	Enable evasion of diagnostic/detection modalities
☐	☐	Enable the weaponization of a biological agent or toxin
☐	☐	Any other potentially harmful combination of experiments and agents

Plants

Seed stocks	*Report on the source of all seed stocks or other plant material used. If applicable, state the seed stock centre and catalogue number. If plant specimens were collected from the field, describe the collection location, date and sampling procedures.*
Novel plant genotypes	*Describe the methods by which all novel plant genotypes were produced. This includes those generated by transgenic approaches,*

Novel plant genotypes	*gene editing, chemical/radiation-based mutagenesis and hybridization. For transgenic lines, describe the transformation method, the number of independent lines analyzed and the generation upon which experiments were performed. For gene-edited lines, describe the editor used, the endogenous sequence targeted for editing, the targeting guide RNA sequence (if applicable) and how the editor was applied.*
Authentication	*Describe any authentication procedures for each seed stock used or novel genotype generated. Describe any experiments used to assess the effect of a mutation and, where applicable, how potential secondary effects (e.g. second site T-DNA insertions, mosiacism, off-target gene editing) were examined.*

ChIP-seq

Data deposition

☐ Confirm that both raw and final processed data have been deposited in a public database such as GEO.

☐ Confirm that you have deposited or provided access to graph files (e.g. BED files) for the called peaks.

Data access links<br>*May remain private before publication.*	*For "Initial submission" or "Revised version" documents, provide reviewer access links. For your "Final submission" document, provide a link to the deposited data.*
Files in database submission	*Provide a list of all files available in the database submission.*
Genome browser session<br>(e.g. UCSC)	*Provide a link to an anonymized genome browser session for "Initial submission" and "Revised version" documents only, to enable peer review. Write "no longer applicable" for "Final submission" documents.*

Methodology

Replicates	*Describe the experimental replicates, specifying number, type and replicate agreement.*
Sequencing depth	*Describe the sequencing depth for each experiment, providing the total number of reads, uniquely mapped reads, length of reads and whether they were paired- or single-end.*
Antibodies	*Describe the antibodies used for the ChIP-seq experiments; as applicable, provide supplier name, catalog number, clone name, and lot number.*
Peak calling parameters	*Specify the command line program and parameters used for read mapping and peak calling, including the ChIP, control and index files used.*
Data quality	*Describe the methods used to ensure data quality in full detail, including how many peaks are at FDR 5% and above 5-fold enrichment.*
Software	*Describe the software used to collect and analyze the ChIP-seq data. For custom code that has been deposited into a community repository, provide accession details.*

Flow Cytometry

Plots

Confirm that:

☒ The axis labels state the marker and fluorochrome used (e.g. CD4-FITC).

☒ The axis scales are clearly visible. Include numbers along axes only for bottom left plot of group (a 'group' is an analysis of identical markers).

☒ All plots are contour plots with outliers or pseudocolor plots.

☒ A numerical value for number of cells or percentage (with statistics) is provided.

Methodology

Sample preparation	C57BL/6J mice after CSDS were deeply anesthetized with pentobarbital sodium (20 mg/kg, i.p.). Subsequently, mice were perfused intracardially with 20 ml of cold Hank's Balanced Salt Solution (HBSS), followed by a rapid collection of the bilateral mPFC, which was washed with cold PBS and chopped into small pieces on ice. Small tissue was mechanically homogenized using 23G needle, producing cell suspensions which were filtered through a 40 µm cell strainer. Subsequently, the samples were strained and centrifuged at 4 ℃ (300 g, 10 min). The pellets were resuspended in 38% percoll solution and centrifuged at 4 ℃ (800g, 10 min). After careful removal of the supernatant, the pellets were rinsed with PBS. The cell surface markers were stained for 30 min at 4℃ with FcR blocking reagent (Miltenyi) and treated with ACSA-2-PE of the FACS antibodies (Miltenyi Biotec, 130-102-365). Data collection was performed on a BD LSRFortessa X-20 and subsequent analysis was completed using FlowJo software.
Instrument	BD LSRFortessa X20
Software	FlowJo

Cell population abundance | At least 10^5 GFP+ cells were collected for further experiments.

Gating strategy | Briefly, FSC-A/ SSC-A were used to identify viable cells. FSC-W/ FSC-H gating was used to identify singlet cells. FSC-H/ ACSA-PE gating was used to identify PE+ cells

☐ Tick this box to confirm that a figure exemplifying the gating strategy is provided in the Supplementary Information.

Magnetic resonance imaging

Experimental design

Design type | *Indicate task or resting state; event-related or block design.*

Design specifications | *Specify the number of blocks, trials or experimental units per session and/or subject, and specify the length of each trial or block (if trials are blocked) and interval between trials.*

Behavioral performance measures | *State number and/or type of variables recorded (e.g. correct button press, response time) and what statistics were used to establish that the subjects were performing the task as expected (e.g. mean, range, and/or standard deviation across subjects).*

Acquisition

Imaging type(s) | *Specify: functional, structural, diffusion, perfusion.*

Field strength | *Specify in Tesla*

Sequence & imaging parameters | *Specify the pulse sequence type (gradient echo, spin echo, etc.), imaging type (EPI, spiral, etc.), field of view, matrix size, slice thickness, orientation and TE/TR/flip angle.*

Area of acquisition | *State whether a whole brain scan was used OR define the area of acquisition, describing how the region was determined.*

Diffusion MRI ☐ Used ☐ Not used

Preprocessing

Preprocessing software | *Provide detail on software version and revision number and on specific parameters (model/functions, brain extraction, segmentation, smoothing kernel size, etc.).*

Normalization | *If data were normalized/standardized, describe the approach(es): specify linear or non-linear and define image types used for transformation OR indicate that data were not normalized and explain rationale for lack of normalization.*

Normalization template | *Describe the template used for normalization/transformation, specifying subject space or group standardized space (e.g. original Talairach, MNI305, ICBM152) OR indicate that the data were not normalized.*

Noise and artifact removal | *Describe your procedure(s) for artifact and structured noise removal, specifying motion parameters, tissue signals and physiological signals (heart rate, respiration).*

Volume censoring | *Define your software and/or method and criteria for volume censoring, and state the extent of such censoring.*

Statistical modeling & inference

Model type and settings | *Specify type (mass univariate, multivariate, RSA, predictive, etc.) and describe essential details of the model at the first and second levels (e.g. fixed, random or mixed effects; drift or auto-correlation).*

Effect(s) tested | *Define precise effect in terms of the task or stimulus conditions instead of psychological concepts and indicate whether ANOVA or factorial designs were used.*

Specify type of analysis: ☐ Whole brain ☐ ROI-based ☐ Both

Statistic type for inference (See Eklund et al. 2016) | *Specify voxel-wise or cluster-wise and report all relevant parameters for cluster-wise methods.*

Correction | *Describe the type of correction and how it is obtained for multiple comparisons (e.g. FWE, FDR, permutation or Monte Carlo).*

Models & analysis

n/a	Involved in the study
☐	☐ Functional and/or effective connectivity
☐	☐ Graph analysis
☐	☐ Multivariate modeling or predictive analysis

Functional and/or effective connectivity	*Report the measures of dependence used and the model details (e.g. Pearson correlation, partial correlation, mutual information).*
Graph analysis	*Report the dependent variable and connectivity measure, specifying weighted graph or binarized graph, subject- or group-level, and the global and/or node summaries used (e.g. clustering coefficient, efficiency, etc.).*
Multivariate modeling and predictive analysis	*Specify independent variables, features extraction and dimension reduction, model, training and evaluation metrics.*
